# Unveiling the human nitroproteome: Protein tyrosine nitration in cell signaling and cancer

**DOI:** 10.1016/j.jbc.2023.105038

**Published:** 2023-07-12

**Authors:** Irene Griswold-Prenner, Arun K. Kashyap, Sahar Mazhar, Zach W. Hall, Hossein Fazelinia, Harry Ischiropoulos

**Affiliations:** 1 Nitrase Therapeutics, Brisbane, California, USA; 2 Children’s Hospital of Philadelphia Research Institute, University of Pennsylvania, Philadelphia, Pennsylvania, USA

**Keywords:** tyrosine nitration, posttranslational modifications, 3-nitrotyrosine, cell signaling, cancer biology

## Abstract

Covalent amino acid modification significantly expands protein functional capability in regulating biological processes. Tyrosine residues can undergo phosphorylation, sulfation, adenylation, halogenation, and nitration. These posttranslational modifications (PTMs) result from the actions of specific enzymes: tyrosine kinases, tyrosyl-protein sulfotransferase(s), adenylate transferase(s), oxidoreductases, peroxidases, and metal-heme containing proteins. Whereas phosphorylation, sulfation, and adenylation modify the hydroxyl group of tyrosine, tyrosine halogenation and nitration target the adjacent carbon residues. Because aberrant tyrosine nitration has been associated with human disorders and with animal models of disease, we have created an updated and curated database of 908 human nitrated proteins. We have also analyzed this new resource to provide insight into the role of tyrosine nitration in cancer biology, an area that has not previously been considered in detail. Unexpectedly, we have found that 879 of the 1971 known sites of tyrosine nitration are also sites of phosphorylation suggesting an extensive role for nitration in cell signaling. Overall, the review offers several forward-looking opportunities for future research and new perspectives for understanding the role of tyrosine nitration in cancer biology.

Tyrosine nitration (Fig. 1*A*) is a covalent posttranslational protein modification derived from both enzymatic and nonenzymatic chemistries (1, 2, 3, 4, 5, 6, 7, 8, 9, 10, 11, 12, 13, 14, 15). The modification entails the addition of a nitro-(NO_2_) group to one of the two equivalent *ortho* carbons of the phenolic ring of tyrosine residues in proteins. Tyrosine nitration and halogenation (addition of chloride or bromide onto the ortho carbon) (Fig. 1*A*) are the newest entries in the biological repertoire of tyrosine posttranslational modifications (PTMs). Modifications targeting the hydroxyl group of the phenolic ring such as sulfation, AMPylation, (referred to as adenylylation) or adenylation and phosphorylation (Fig. 1*A*) were described in the 1960s and 1970s and have emerged as important regulators of protein function and signal transduction (16, 17, 18, 19, 20, 21, 22, 23, 24, 25, 26, 27). Initially tyrosine nitration and halogenation were employed only as biochemical tools to study protein function and structure (28). Exploration of tyrosine nitration as a biochemical approach to study proteins has been driven primarily by three considerations: (i) tyrosine is present in most proteins, comprising on average 3.2% of amino acid residues. (ii) the amphipathic nature of the phenolic ring enables its localization both in hydrophobic cores and on the protein surface, often at interfaces of proteins with small molecules, or other proteins. (iii) the aromatic ring is highly reactive.

**Figure 1:**
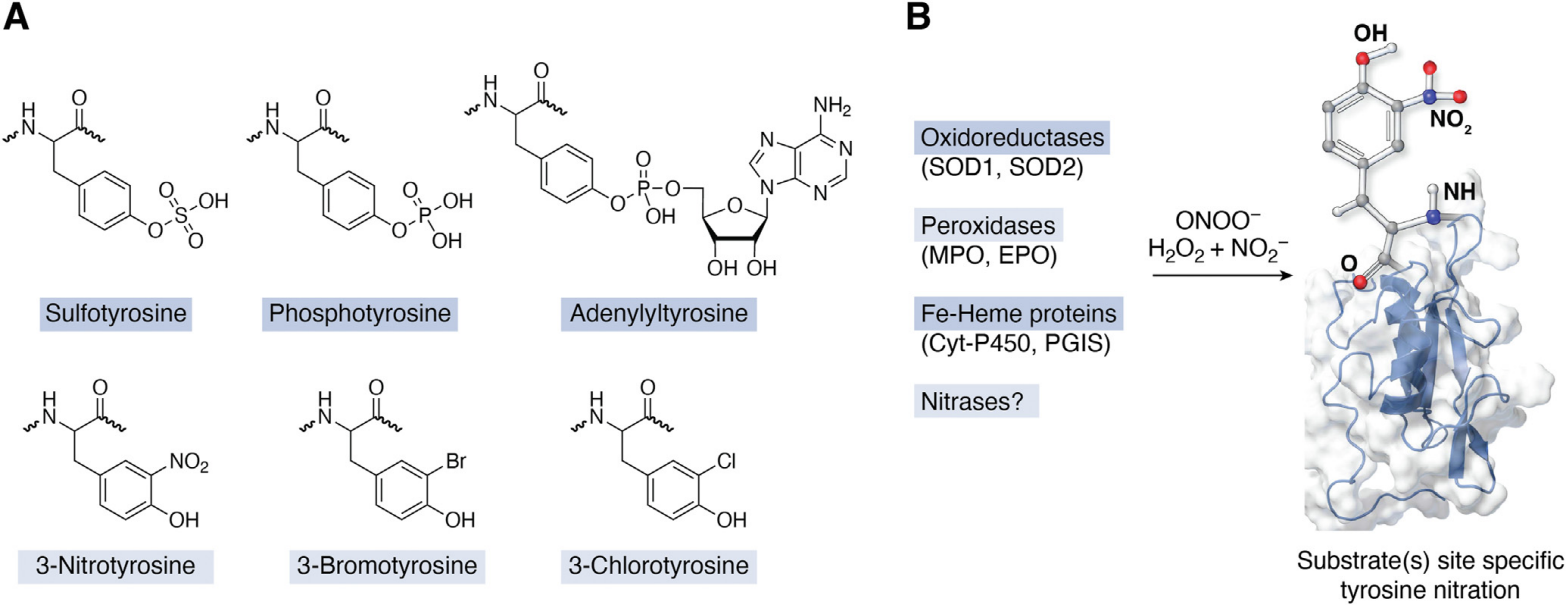
**Tyrosine modifications.***A*, tyrosine modifications targeting the hydroxyl group of the phenolic ring are sulfation, phosphorylation, and AMPylation (referred to as adenylation). Tyrosine nitration and halogenation (addition of chloride/bromide onto the ortho carbon) are the newest entries in the biological repertoire of tyrosine PTMs. *B*, biological pathways and chemistry that contributes to tyrosine nitration. PTMs, posttranslational modifications.

The concept of biologically relevant protein tyrosine nitration and halogenation was not introduced until the early 1990s and coincided with studies seeking to understand the biological chemistry of peroxynitrite (ONOO^−^/ONOOH), the reaction product of two radical species superoxide and nitric oxide (29, 30), as well as the biotransformation of nitrite by peroxidases (6, 7, 8, 9). Nitric oxide converted into reactive nitrating species provides the source of nitrogen. Originally, work was centered on peroxynitrite-based mechanisms that included metal catalysis, the reaction of peroxynitrite with bicarbonate to generate the potent nitrating species, nitroso-peroxocarbonate (ONOOCO_2_^−^), catalysis by metalloproteins and hemeproteins (Fig. 1*B*). A second well studied pathway is the catalysis of protein nitration by the leukocyte enzymes myeloperoxidase (MPO) and eosinophil peroxidase (EPO). These enzymes utilize hydrogen peroxide (H_2_O_2_) and nitrite (NO_2_^−^) to generate nitrating intermediates. Data from multiple distinct models of inflammation using mice deficient in either MPO or EPO confirmed the contribution of MPO and EPO to *in vivo* formation of nitrated proteins (7, 9). However, in some of the leukocyte-rich acute inflammatory models neither MPO nor EPO contributed to protein tyrosine nitration (9). Another plausible mechanism for protein nitration is the formation of nitrous acid from the acidification of nitrite. Although this pathway requires a significant drop in pH (pH < 6.0), accumulation of 3-nitrotyrosine has been observed without an apparent need for superoxide or hydrogen peroxide (31). Detailed description and discussion of the chemistry underpinning the mechanisms of biological tyrosine nitration have been published in previous comprehensive reviews (32, 33). Collectively the published work confirms the existence of multiple biological reactions stemming from the formation of nitric oxide that account for the *in vivo* formation of protein tyrosine nitration.

## Updated overview of protein tyrosine nitration

The initial studies on biological tyrosine nitration spurred a rush of publications that provided tools and knowledge for the quantification and detection of tyrosine nitration in humans and animal models of disease. The development of antinitrotyrosine antibodies, polyclonal and monoclonal antibodies that recognize 3-nitrotyrosine globally (34, 35, 36), was a major first step in exploring protein nitration in biological systems. In addition to challenges relating to specificity, avidity, and affinity of these antibodies, the potential formation of nitrated tryptophan was a confounding factor. Reactive nitrating intermediates also react with cysteine, methionine, histidine, and tryptophan residues leading to the formation of oxidation products (37). Regarding tryptophan, formation of 6-nitrotryptophan as the major derivative and 4- and 5-nitrotryptophan as additional lower yield products has been documented *in vivo* (38, 39). These challenges necessitated the judicious use of negative controls such as pre-absorption with 3-nitrotyrosine or tyrosine nitrated peptides to describe the cellular localization of nitrated proteins at the light and electron microscopic level under physiological conditions, in several human disorders and experimental models of disease (34, 35, 36). Another major advance was the generation of antibodies that precisely detected one or more specific nitrated tyrosine residues in particular proteins. The development, characterization, and use of antibodies that detected specific nitrated tyrosine residues in Mn superoxide dismutase (SOD) 2 sarcoplasmic endoplasmic reticulum (ER) calcium ATPase (SERCA2) (40), and proteins relating to neurodegeneration such as α-synuclein (41), amyloid beta (42), and tau (43) have all confirmed site-specific nitration of these proteins in the context of human disease. Analytical methods for the quantification of 3-nitrotyrosine based on stable isotope dilution mass spectrometric detection quantified protein nitration in human studies as well as in animal and cellular model systems (44). As with the detection of protein phosphotyrosine (45), the detection of nitrated peptides by LC-MS/MS after typical trypsin, or the detection of other enzymatic digestion of proteins has been a challenge due to low abundance and substochiometric levels of nitrated tyrosine residues in cellular and tissue proteomes. To overcome these challenges affinity enrichment approaches using antibodies or peptides have been developed and enabled the site-specific detection of nitrated tyrosine residues in complex cellular and tissue extracts. Inclusion of manual inspection and validation of peptides detected by mass spectrometry /mass spectrometry have also overcome issues relating to potential misidentification of tyrosine nitration. These methodological platforms have been employed to identify, localize, and quantify tyrosine nitration (46, 47, 48, 49, 50, 51, 52, 53, 54, 55, 56, 57, 58, 59, 60, 61, 62, 63). Many of the published studies concluded that tyrosine nitration should be considered as a disease biomarker (64, 65, 66, 67, 68, 69, 70, 71). The causal association with disease pathogenesis and mechanisms is debated and remains an active area of investigation.

Significant new insights into the functional importance of tyrosine nitration of proteins have been gleaned through studies that used the incorporation of the unusual amino acid 3-nitrotyrosine into expressed proteins and by detailed structural and biochemical analysis of nitrated proteins. The generation of proteins with site specific incorporation of 3-nitrotyrosine has been accomplished either by using engineered aminoacyl tRNA synthetases that are specific for 3-nitrotyrosine or by expressed protein ligation methods (72, 73, 74, 75, 76, 77). These technologies, which can generate proteins with specific nitrated tyrosine residues in the absence of other modifications, have led to increased understanding of the stoichiometry of modification and of the specific role of nitration in protein function.

Tyrosine nitration often results in loss of activity but gain of function has been reported for some nitrated proteins (78, 79, 80, 81, 82, 83, 84). The addition of the nitro group to the carbon adjacent to the hydroxyl group lowers the pKa of the tyrosine residue enabling the nitrated residues to have opposite biochemical properties, that is, both decreased hydrophobicity in the protonated form and increased polarity in the unprotonated (anionic) form. These and other changes contribute to the alteration of protein function, stability, and protein-protein interactions (78, 79, 80, 81, 82, 83, 84). Intriguing functional consequences of protein nitration, for example, are the increased rate of self-assembly of fibrinogen during the processes of productive (fibrin) (81) and the apparent nonproductive formation of amyloid-like α-synuclein fibrils (82).

Early on it was also appreciated that not all proteins with tyrosine residues and not all tyrosine residues in a specific protein are nitrated either *in vivo* and *in vitro* (85). As the inventory of proteins with specific tyrosine nitrated residues has increased, several bioinformatic approaches have been developed which attempted to elucidate primary sequence preferences and structural elements that guide *in vivo* tyrosine nitration (85, 86, 87, 88, 89). Analogous to bioinformatic tools developed for predicting phosphorylation sites and the identification of tyrosine kinases, the studies listed in Table 1 have provided computational approaches to the study of protein tyrosine nitration. Starting with the pioneering development of group-based prediction system for tyrosine nitration (GPS-YNO_2_), these computational tools have predicted sites of nitration, and have explored potential motifs as well as biochemical and biophysical properties that may govern the selective nitration of tyrosine residues in proteins.

**Table 1:** Computational approaches developed for studying protein tyrosine nitration

Name	Webpage-details	Year	Reference
GPS-YNO2	http://yno2.biocuckoo.org/index.php	2011	(183)
iNitro-Tyr	http://app.aporc.org/iNitro-Tyr/.	2014	(184)
DeepNitro	http://deepnitro.renlab.org.	2018	(149)
NTyroSite	https://biocomputer.bio.cuhk.edu.hk/NTyroSite/.	2018	(185)
rPTMDetermine	https://pubs.acs.org/doi/abs/10.1021/acs.analchem.0c02148	2020	(186)
PredNTS	http://kurata14.bio.kyutech.ac.jp/PredNTS	2021	(187)
PreNitro	http://103.99.176.239/PredNitro	2022	(188)

In part the biological selectivity of protein nitration has been attributed to self-catalyzed nitration of oxidoreductases, heme and non-heme–metal containing proteins (Fig. 1*B*), the local environment of selective tyrosine residues, and the proximity of the protein to nitric oxide synthases (the source of nitric oxide and which provides the nitrogen for the nitro group) and/or peroxidases that catalyze the enzymatic generation of nitration of L-tyrosine and tyrosine residues on proteins in the presence of hydrogen peroxide and nitrite (1, 2, 4, 5, 6, 7, 8, 9, 10, 11, 12, 13, 14, 15, 31, 34, 90, 91). However, the possibility that additional enzymes can catalyze protein specific tyrosine nitration comparable to tyrosine kinases and sulfatases has not been considered. Research is ongoing to identify enzymes, which we refer to as nitrase, that may specifically execute tyrosine nitration on specific protein substrates, similarly to phosphorylation of protein substrates by tyrosine kinases.

Several studies support the idea that protein tyrosine nitration can affect cell signaling either directly or by interfering with other tyrosine PTMs (92, 93, 94, 95, 96, 97, 98, 99, 100, 101, 102, 103, 104, 105, 106, 107, 108, 109, 110, 111, 112, 113). Tyrosine nitration can change the conformation, localization, and function of proteins, thus fulfilling some of the most basic requirements for a signaling moiety. In addition, several studies have proposed that protein tyrosine nitration is reversible, possibly *via* enzymes like tyrosine phosphatases (114, 115, 116, 117, 118, 119, 120, 121, 122, 123, 124). The dynamic nature of tyrosine nitration has also been demonstrated within mitochondria and cells (114, 115, 116, 117, 118, 119, 120, 121, 122, 123, 124). However, the data remain incomplete as the definite documentation of a bonafide denitrase, that is, an enzyme that removes the nitro group without degrading the protein, awaits confirmation. Removal of nitrated proteins by various cellular proteolytic systems has been clearly demonstrated (125, 126, 127). Alternatively, nitrated proteins may be removed *via* immunological processes. This suggestion is supported by data indicating that tyrosine nitrated peptides elicit robust immune responses in transgenic mice incapable of mounting a response to non-nitrated tyrosine containing peptide (128, 129). Furthermore, circulating antibodies that recognize nitrated peptides and proteins have been reported in humans (130, 131, 132).

Overall, tyrosine nitration may impact functional protein networks and signaling in disease by altering protein function, localization, turnover, and protein-protein interactions. The potential roles of protein tyrosine nitration in cancer biology and signaling are outlined and discussed in this review.

## Generation of a human tyrosine nitrated protein database

**Figure 2:**
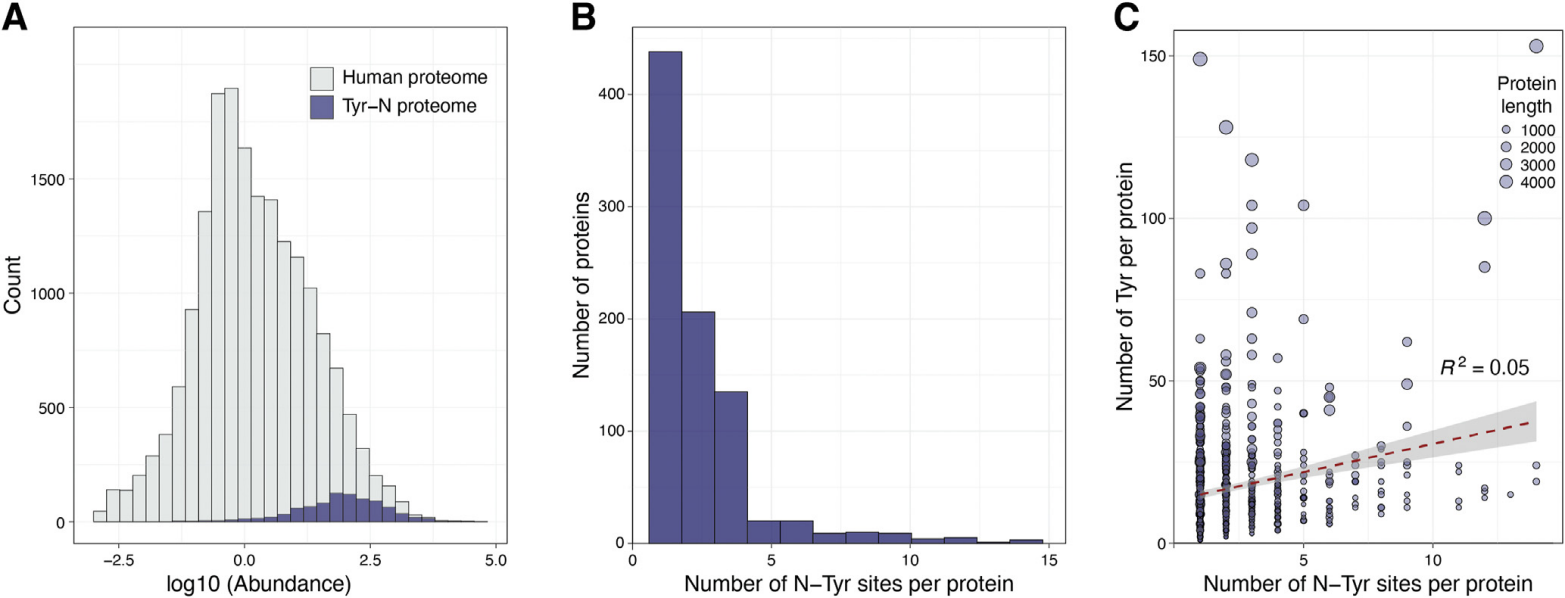
**Tyrosine nitrated proteins.***A*, cumulative frequency of the relative abundance of the 908 tyrosine-nitrated proteins (*purple*) using an integrated 18,694 human protein abundance histogram as background (*gray*). *B*, the distribution of the number of tyrosine nitration sites per protein. The distribution was created by including all the 860 unique proteins (both canonical and isoforms) and the localized 1971 sites. *C*, linear regression analysis between the number of nTyr residues and the number of Tyr residues per protein. The size of the *circle* indicates the number of amino acids per protein (length).

Using all available resources (for a detailed description for methodologies applied see Box 1 and Box 2) we have unearthed 908 human proteins that are or could be nitrated on tyrosine residues. We utilized the curated list of nitrated proteins to explore the distribution of nitrated proteins in terms of their relative abundance in the human proteome. To generate this distribution plot (Fig. 2*A*), we uploaded the most current (2021) relative abundance integrated human proteome data from the PAXdb: Protein Abundance Database (133). We then mapped and curated the data using UniProtKB (134) to generate a list of relative abundance for 18,694 human proteins. The data in Figure 2*A* depict the distribution of the reported nitrated proteins in the human proteome. Of the 908 proteins only 19 did not have a numerical value. The abundance of the 889 nitrated proteins spanned at least seven orders of magnitude from relative abundance of 0.046 for the voltage-dependent L-type calcium channel subunit alpha-1C to 30,702 for Apolipoprotein A-II. Overall, most of the nitrated proteins clustered toward relatively abundant proteins in the human proteome.

Box1 **Creation of an updated database of currently reported tyrosine nitrated human proteins. The database is included as a searchable excel file in Supporting Information.**

**Figure:**
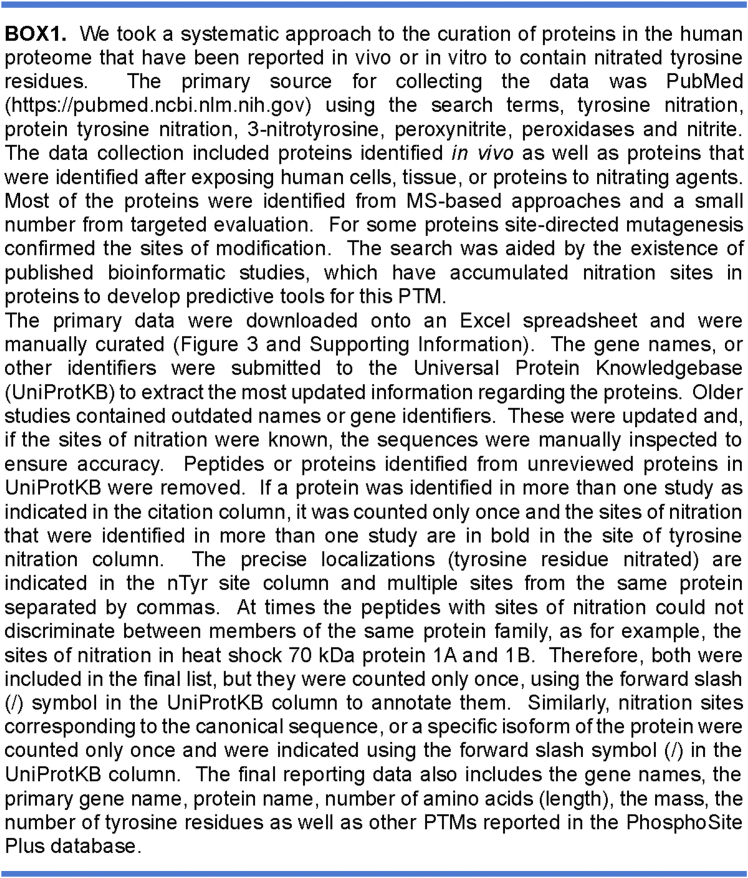

Box2 **Flow chart for the acquisition, curation, and analysis of the current human tyrosine nitrated proteins.**

**Figure:**
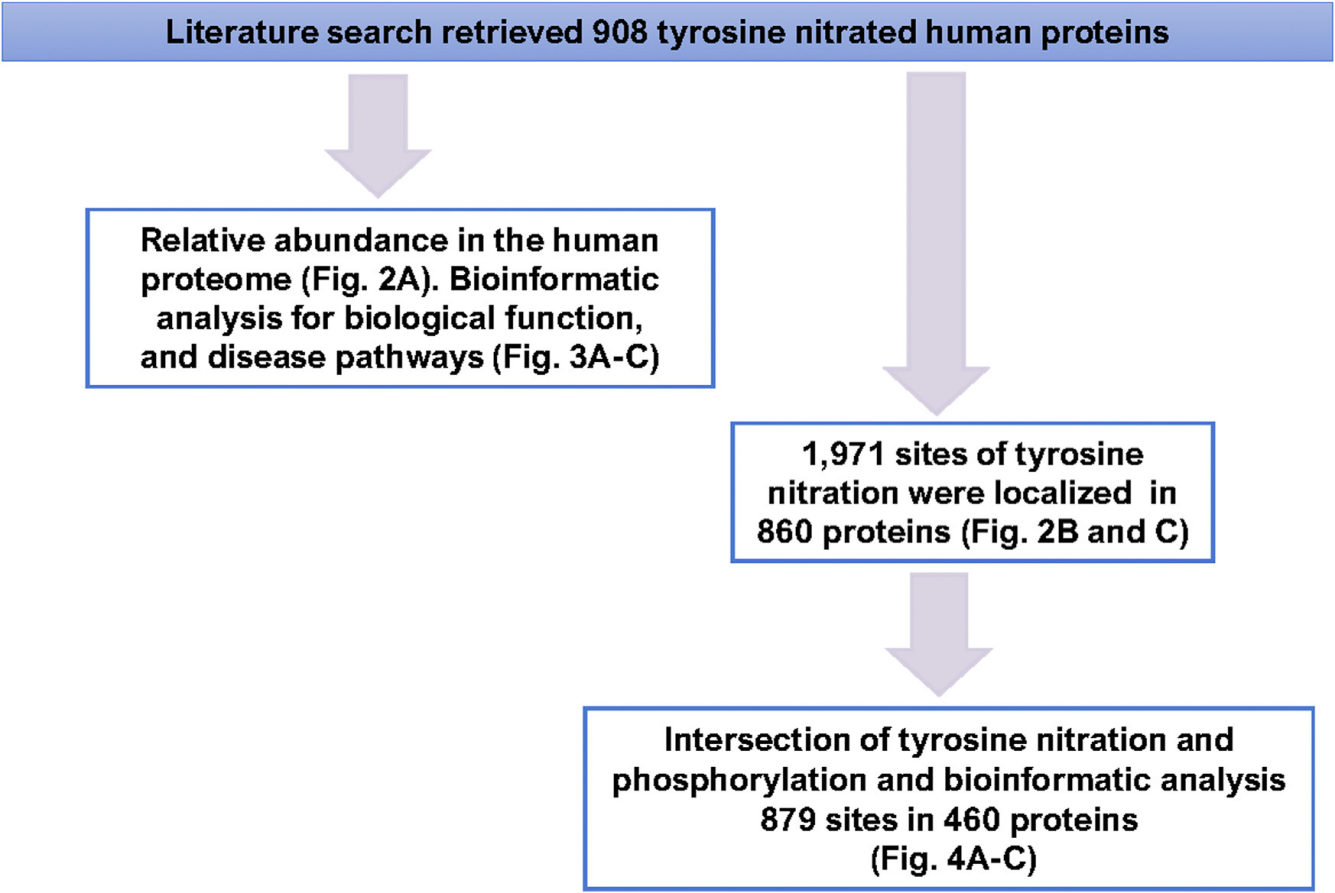

Of the 908 proteins, 860 had known sites of tyrosine nitration totaling 1971 residues, which represent 13.5% of the 14,675 tyrosine residues in these proteins. Nitration sites ranged from 1 to 14 in each protein. Most (21.9%) had a single nitration site whereas, 10.5%, 4.3%, and 2.5% of the nitrated proteins had 2, three or four sites, respectively, (Fig. 2*B*). Figure 2*C* depicts the frequency distribution of nitrated tyrosine residues as a function of the total number of tyrosine residues in the proteins. The data indicated that the occurrence of tyrosine nitration is independent of the total number of tyrosine residues in a protein (R^2^ = 0.05, N = 860), length, or the total number of amino acids (R^2^ = 0.0256). Similarly, there is no apparent correlation between number of nitration sites and either molecular weight of the proteins (R^2^ = 0.0247), or relative abundance (R^2^ = 0.0427). These observations are consistent with previous data indicating that not all proteins with tyrosine residues and not all tyrosine residues in proteins are amenable to nitration. Both experimental as well as computational and structural analyses have reaffirmed that tyrosine nitration is a selective and determined PTM akin to other tyrosine modifications.

## Functional analysis of the human tyrosine nitrated proteins

**Figure 3:**
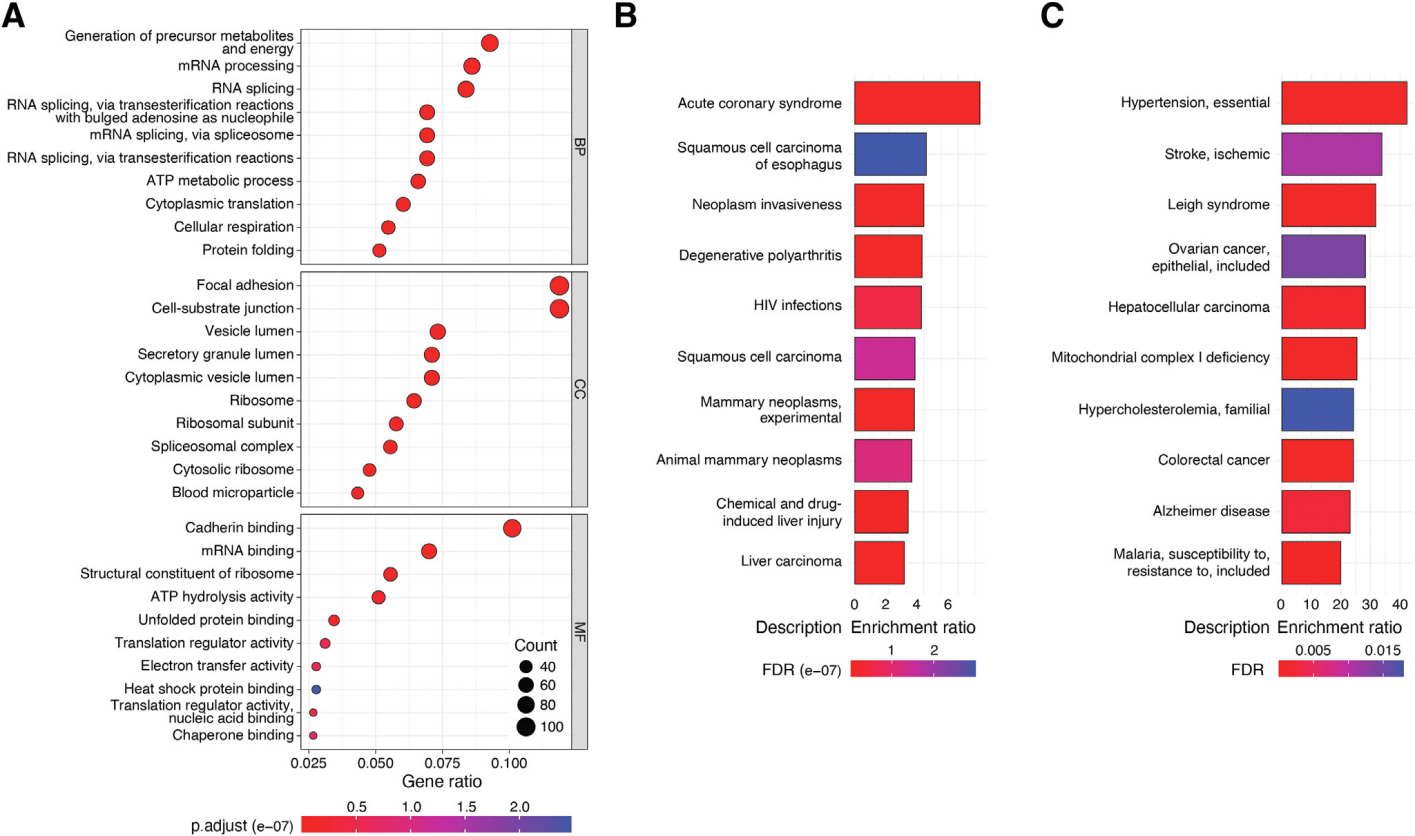
**Analysis of tyrosine-nitrated proteins.***A*, functional analysis of the 908 tyrosine-nitrated proteins using the gene ontology data base. The data depicts the top ten annotations for biological processes (BP), cellular component (CC), and molecular function (MF) considering the values for false discovery rate (values are indicated by the heat map) and fold enrichment. The size of the *circle* indicates the number of genes annotated in the corresponding BP, CC, and MF. *B*, bioinformatic analysis of the 908 tyrosine-nitrated proteins for association (over-representation) with human disorders, using DisGeNET, a collection of genes and gene variants associated with human disease or (*C*), the online catalog of Human Genes and Genetic Disorders (https://www.omim.org). The top ten human associated disorders based on the false discovery rate (FDR) values are shown.

The primary gene names for the 908 proteins were submitted to Gene Ontology (135) and analyzed for biological processes, cellular component (localization), and molecular function enrichment. This analysis revealed three major cellular networks that could be impacted by tyrosine nitration. These include pathways for protein synthesis, protein folding, and metabolism (Fig. 3*A*). Proteins participating in mRNA processing, mRNA binding, RNA splicing, proteins with ribosomal location as well as proteins in spliceosomal complexes are significantly enriched within the 908 proteins. The clustering of nitrated proteins in the pathways of protein synthesis is novel and merits further consideration. Proteins that participate in protein folding included several known chaperones such as heat shock 70 kDa protein 1A and 1B and associated interactors DnaJ homolog subfamily A member 2 and DnaJ homolog subfamily B member 1, heat shock cognate 71 kDa protein, heat shock protein (HSP) 90-alpha and -beta (HSP90), protein disulfide isomerase (PDI)A3, and peptidyl-prolyl *cis*-*trans* isomerase B. Although the functional roles of tyrosine nitration in these proteins remain vastly unknown, emerging data that are discussed below indicates both loss of chaperone function and a gain of function of HSP90 by selective tyrosine nitration. The gain of HSP90 function relates to mitochondrial bioenergetics. Nitration of mitochondrial protein participating in the tricarboxylic acid cycle, electron transport chain (ETC), primarily of cytochrome c (14), and antioxidant defenses (78, 79, 80) have been implicated in reduced mitochondrial functional capacity and disruption of metabolism. Nitration of three key proteins, 60 kDa HSP, 10 kDa HSP, and stress-70 protein that facilitate mitochondrial protein import and macromolecular assembly of ETC complexes and biogenesis of mitochondrial iron-sulfur cluster may also profoundly influence mitochondrial function. Further work is needed to assess the role of tyrosine nitration in these three mitochondrial proteins.

To appreciate the potential biological implications of protein tyrosine nitration, we utilized known web-based bioinformatic tools that execute functional enrichment analysis such as the Kyoto Encyclopedia of Genes and Genomes (KEGG), WEB-based Gene SeT AnaLysis Toolkit (WebGestalt) and Enrichr (136, 137, 138). Functional and disease related enrichment across these platforms returned expected data that associate protein nitration with neurodegenerative, cardiovascular, and metabolic disorders. A new insight regarding the association of protein nitration and human disorders emerged from using either the DisGeNET (139) or Online Catalog of Human Genes and Genetic Disorders (OMIM) two discovery platforms that associate genes with human disease. This analysis revealed that tyrosine nitration may be associated with different types of cancer, by altering metabolic reprograming and signaling (Fig. 3, *B* and *C*). Although the potential roles of tyrosine nitration in neurodegeneration, cardiovascular, and metabolic diseases have been reviewed previously (140, 141, 142, 143, 144, 145, 146, 147), the implications in cancer biology merit further consideration and are highlighted in this review.

**Figure 4:**
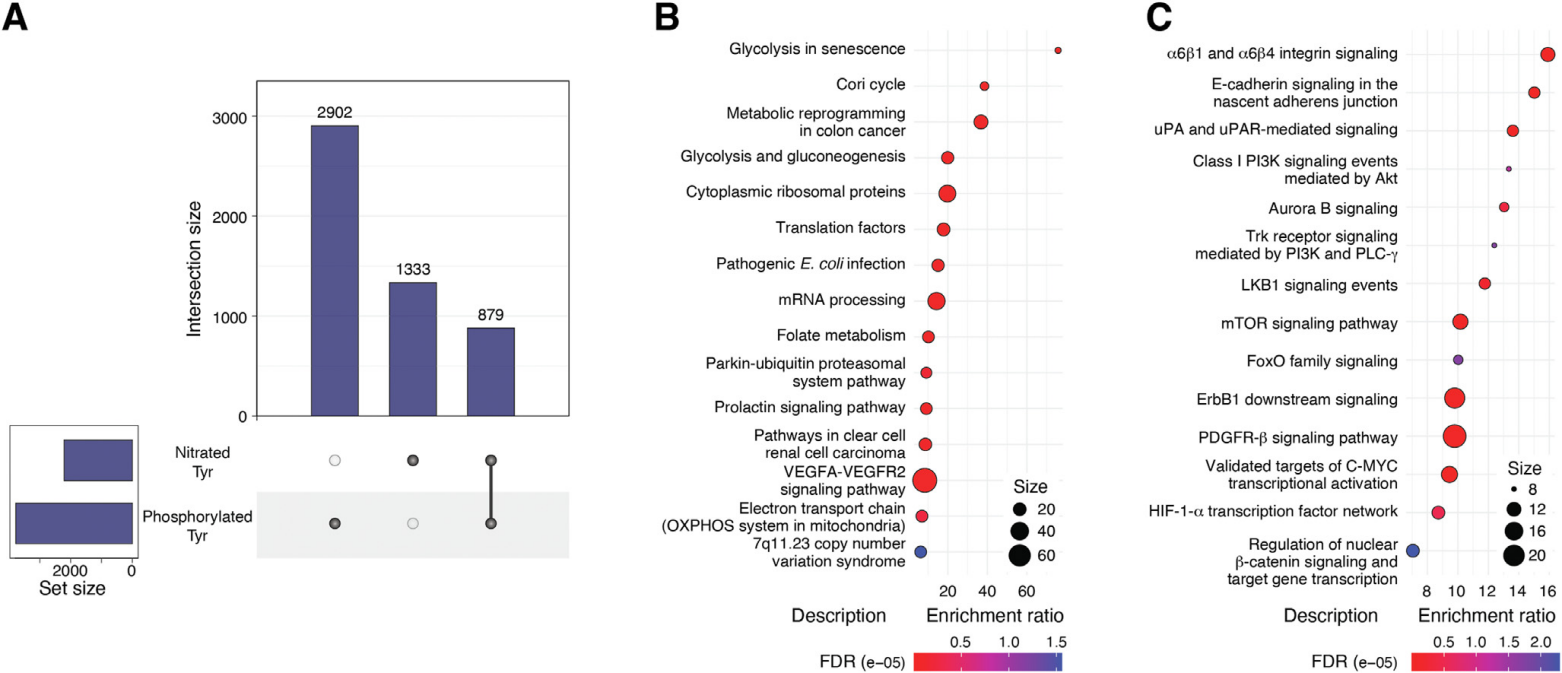
**Intersection of tyrosine nitration and phosphorylation.***A*, upset plot for the intersection of tyrosine nitration and tyrosine phosphorylation. *B* and *C*, bioinformatic analysis of the 460 tyrosine-nitrated proteins that shared one or more tyrosine phosphorylation and nitration sites for signaling pathways. The primary analysis utilized Enrichr and either the Wiki Pathways (*B*) or the National Cancer Institute Nature discovery (*C*) platforms. The top categories based on the FDR values are depicted. The size of the bubble indicates the number of genes in the cluster. FDR, false discovery rate.

We also explored the intersection between tyrosine nitration and tyrosine phosphorylation in this new dataset. We utilized the wed-based tool PhosphoSitePlus (148) to explore other PTMs in the 860 proteins with known sites of tyrosine nitration and specifically tyrosine phosphorylation (Fig. 4*A* and Supporting Information). Interestingly, nitration and phosphorylation overlapped in 879 tyrosine residues in 460 proteins (Fig. 4*A*). The 460 proteins had a total of 7798 tyrosine residues of which 2902 (37.2%) have been annotated as phosphorylated and 1333 (17.1%) as nitrated sites. The 879 overlapping sites represent 30.3% of the phosphorylation events in these proteins. The overlap was not restricted to a single tyrosine residue as proteins with up to 12 overlapping phosphorylated and nitrated proteins were identified. This new revelation suggests a potential interplay between tyrosine phosphorylation and nitration far beyond what was expected. An overlap between tyrosine nitration and phosphorylation was also reported previously (149) utilizing a smaller set of tyrosine nitrated proteins from all species. Given that the frequency of both modifications is relatively low and potentially underestimated, tyrosine nitration could profoundly interfere or alternatively complement tyrosine phosphorylation. Published studies indicate that tyrosine nitration and phosphorylation are mutually exclusive presumably due to the deactivation of the aromatic ring reactivity by one or the other modification. In part, this overlap could be explained by the presence of surface exposed tyrosine residues that are accessible to tyrosine kinases for the transfer of phosphate. While surface exposure is a key regulatory element of tyrosine nitration, the observation also suggests that tyrosine nitration may be facilitated by enzymes that utilize peroxynitrite, or nitrite and hydrogen peroxide to transfer the nitro group onto tyrosine residues. This opens the possibility that there exists a new class of enzymes, the nitrases, that control site specific tyrosine nitration (Fig. 1*B*) in the same way that tyrosine kinases control tyrosine phosphorylation in signal transduction pathways. Using WikiPathways (150) and the National Cancer Institute-Nature pathway interaction databases (151) several functional and signaling pathways may be impacted by the overlapping tyrosine nitrated and phosphorylated tyrosine residues in the 460 proteins (Fig. 4, *B* and *C*).

The relationship between aberrant activation of growth and survival signaling pathways in cancer *via* increased tyrosine phosphorylation of proteins such as epidermal growth factor receptor (EGFR), proto-oncogene tyrosine-protein kinase SRC (SRC) family, anaplastic lymphoma kinase and tyrosine-protein kinase Met has been well established and serve as the basis for numerous targeted therapies (152). The data reveal that many of the sites that are hyperphosphorylated, and serve as drivers in cancer, overlap sites of nitration. Notable proteins that showed overlap include EGFR, growth factor receptor–bound protein 2 (GRB2), lactate dehydrogenase A chain (LDHA) and phosphatase 1B (PTP1B). The biological relevance of nitration in the proteins mentioned above has yet to be elucidated but offers an exciting new area of investigation. However, there also exists an impressive body of work that describes the functional consequences and, in some cases, tumor promoting roles of site-specific nitration in several proteins. Below we highlight these central findings.

## Protein nitration in oncology

**Figure 5:**
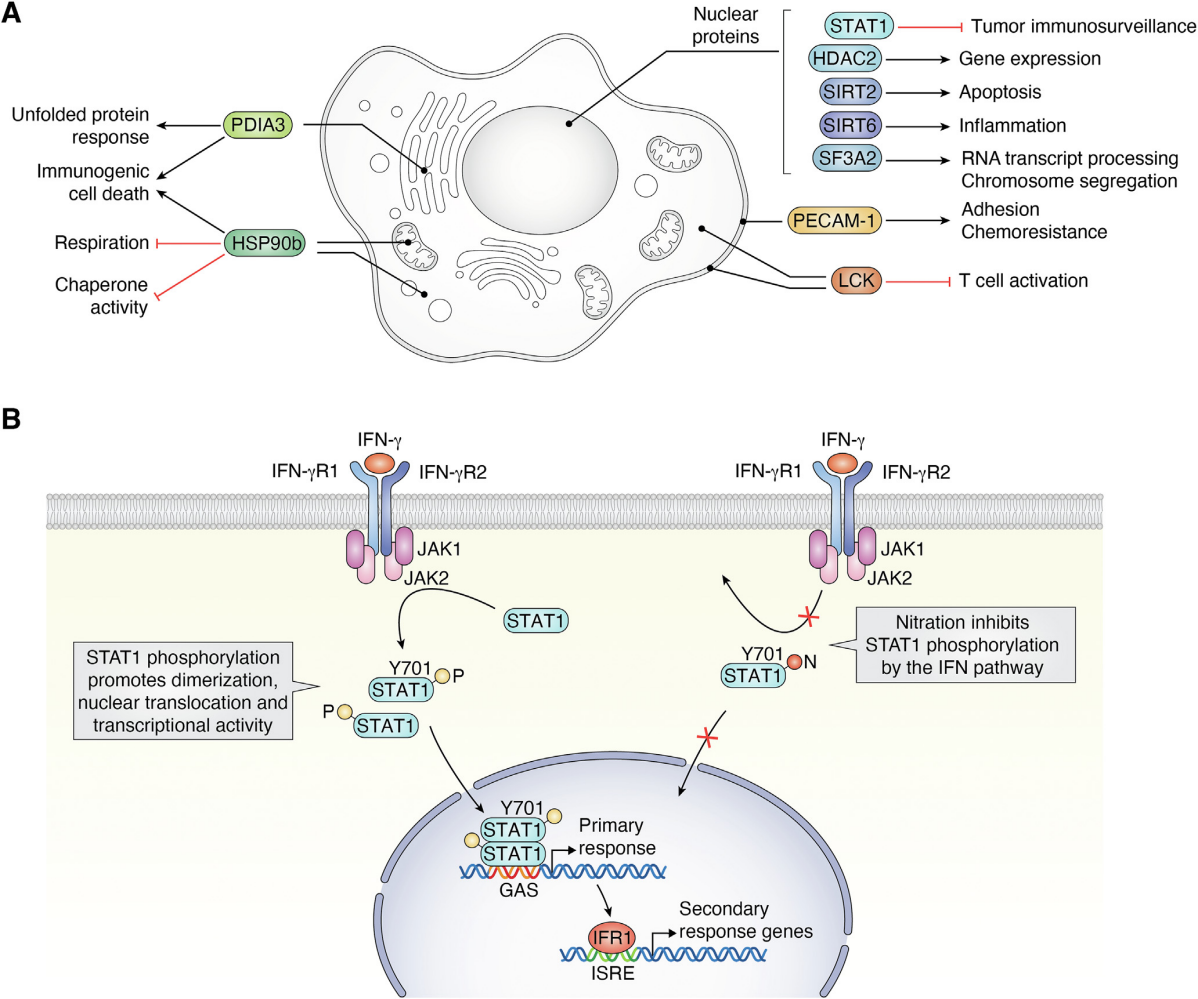
**Summary of nitrated proteins implicated in oncology.***A*, nitrated proteins implicated in oncology include those involved in intracellular signaling, immune surveillance, cytokine pathway stimulation, Damage Associated Molecular Pattern (DAMP) expression, and resistance to chemotherapeutics. Nitration of proteins can serve a regulatory function, and the tyrosine residues that are nitrated in many of these proteins are often the same as those that are tyrosine phosphorylated. Nitration of some inhibit tyrosine phosphorylation to regulate protein activity. *B*, STAT1 nitration inhibits its phosphorylation and signaling. The interferon (IFN) pathway is essential for immunosurveillance. IFN activates immunosurveillance by phosphorylation at tyrosine (Y701) of the signal transducer and activator of transcription 1-alpha/beta (STAT1). STAT1 phosphorylation at (Y701) is required for translocation to the nucleus to activate transcription of its target genes. Nitration at tyrosine (Y701) inhibits STAT1 phosphorylation, translocation, and activation of transcription.

A significant subset of the proteins found in the database of human nitrated proteins are associated with cancer (Fig. 5*A*). This class includes those involved in intracellular signaling, immune surveillance, cytokine pathway stimulation, damage-associated molecular pattern expression and resistance to chemotherapeutics. For several, nitration of the protein is known to serve a regulatory function. The tyrosine residues that are nitrated in many of these proteins are often identical to sites of phosphorylation, and the nitration of some is known to inhibit phosphorylation modifications that regulate protein activity. We provide here several examples of proteins related to cancer that are nitrated. For this purpose, we have restricted the examples to proteins in which the specific tyrosine that is nitrated is known and in which there is clear evidence to connection to pathways known to play a role in oncology.

## Interferon and STAT1 signaling in immunosurveillance

The interferon (IFN) pathway is essential for immunosurveillance of potential tumor cells. IFN is known to activate the immunosurveillance by tyrosine phosphorylation of the signal transducer and activator of transcription 1-alpha/beta (STAT1). Phosphorylation of a specific tyrosine (Y701) is required for translocation of STAT1 to the nucleus to activate transcription of its target genes. Reduced activation of this pathway has been found in immune effector cells from patients with advanced tumors.

Experiments in mice and human cells have found that STAT1 can be nitrated at Y701, thus reducing phosphorylation at this position, and that this nitration is increased in specific cancers (Fig. 5*B*) (153). CD4^+^ and CD8^+^ splenic cells from mice bearing the C26 carcinoma, which have reduced immunosurveillance, have been found to have reduced levels of IFNa and IFNg-induced phosphorylation of STAT1 Y701, suggesting that nitration might be the mechanism that impairs immunosurveillance in these cells.

Consistent with this hypothesis, splenocytes from C-26-tumor-bearing mice have increased nitration of STAT1, as well as increased levels of the inducible isoform of nitric oxide synthase (iNOS and NOS2) and nitric oxide. Increased nitration of Y701 has also been observed in peripheral blood mononuclear cells from eight melanoma and eight pancreatic cancer patients when compared to nine nondisease controls (153). These experiments suggest that immunosurveillance may be reduced in certain cancers associated with elevated nitric oxide levels by increased nitration of STAT1 Y701, thus decreasing phosphorylation at that site, and inhibiting the IFN-STAT-1 signaling cascade (Fig. 5*B*).

## Tyrosine nitration of lymphocyte-specific protein tyrosine kinase (LCK) in T-cell tolerance

Tyrosine nitration at a specific tyrosine (Y394) in LCK, which is a major, initiating contributor of T cell receptor signaling, has also been observed in tumor-infiltrating T-lymphocytes (154). Nitration is increased in anti-CD3/CD28-stimulated splenic T cells in the presence of activated myeloid-derived suppressor cells (MDSCs) which impair immunosurveillance. In a separate experiment, MDSCs were found to generate increased reactive nitrogen species in both a transgenic model of prostate cancer and in a syngeneic cell line model of lung cancer. Moreover, nitration of Y394-LCK was found to inhibit LCK activity and T-cell activation, resulting in reduced interleukin 2 production and T cell proliferation. Thus, diminished T-cell proliferation and activation resulting from the inhibition of LCK activity through nitration of Y394 provides a second mechanism by which immunosurveillance of cancers may be reduced.

These STAT1 and LCK data suggest that inhibition of either STAT1 or LCK nitration could be effective mechanisms for enhancing effects of immune-oncology therapies.

## Tyrosine nitration of JAK2

Growth hormone induces nitration of Janus Kinase 2 (JAK2) at nY1007/1008 (106). These are amino acids that are also typically phosphorylated to activate JAK2. Nitration of these JAK2 amino acids inhibits the subsequent phosphorylation and activation of the protein.

Elsasser *et al.* (106) demonstrated that a lipopolysaccharide challenge in calves resulted in JAK2 nitration and reduction in JAK2 levels in a liver biopsy. This suggests that inflammatory states regulate nitration and thereby levels and activity of JAK2. Given JAK2’s role as an oncogene, its association with liver cancer and the causal role described for JAK2 in models of liver cancer (155, 156), one can postulate that nitration of JAK2 is also found in, and contributes to, liver cancer progression.

## Nitration of the regulatory subunit of protein phosphatase 2A and anti-apoptosis

B56delta (B56δ) is a regulatory subunit of protein phosphatase 2A (PP2A) that imparts substrate specificity. One of the substrates of PP2A-B56δ is B-cell lymphoma 2 (BCL2), a protein that is overexpressed in hematopoietic malignancies. The antiapoptotic activity of BCL2 is regulated by phosphorylation of a specific serine residue (S70) (157). This site is normally dephosphorylated by the PP2A-B56δ holoenzyme thus inhibiting BCL2 (158).

Under increased oxidative stress, B56δ becomes nitrated at a specific tyrosine (Y289) (159). In Jurkat cells, the nitrated form of B56δ can no longer bind to the PP2A catalytic core to dephosphorylate BCL2, leading to hyperphosphorylation of BCL2 at S70 and resistance to chemotherapy-induced apoptosis. Using an antibody generated specifically against nitrated B56δ, the authors showed increased levels of B56δ nitration in lymphoma biopsy samples as well as corresponding hyperphosphorylation of BCL2 specifically in samples with high nitrated B56δ. In Jurkat cells, inhibition of B56δ nitration using redox modulators such as FeTPPS led to restoration of PP2A holoenzyme formation, BCL2 dephosphorylation, and sensitivity to chemotherapy. The development of a targeted approach to inhibit BCL2 nitration thus may represent a unique therapeutic opportunity in these malignancies.

## Nitration of growth factors and chemokines

Neuregulin 1 (NRG1) activates the human receptor tyrosine-protein kinase erbB-2 (HER2), which is overexpressed in breast, ovarian, bladder, pancreatic, stomach, and esophageal cancers. Experiments in A549 lung cancer cells have shown that iNOS is induced upon stimulation with cytokines, resulting in the nitration of NRG1 at residues Y208, Y224, and Y230 (160). Nitration of these residues produced a decrease in the ability of NRG1 to induce phosphorylation and activation of HER2. Nitration of NRG1 abolished the binding of antibodies that recognize the epidermal growth factor-like domain of NRG1, apparently interfering with the binding of NRG1 to HER2. The data suggests that in a proinflammatory state, NRG1 can be nitrated, thus inducing a nonfunctional state with reference to activation of the HER2 pathway and subsequent proliferation. Understanding more about how nitration regulates NRG1 could shine additional light on understanding of the role that nitration and oxidative/nitrative stress play in regulation of the HER2 pathway.

## CCL2 and CXCL12: leukocyte chemotaxis

Leukocyte chemotaxis is driven by response to chemokines, particularly CCL2 and CXCL12. Both chemokines are nitrated by peroxynitrite, and both show reduced binding to their receptors as a consequence (161, 162). Conflicting data have been reported for the effects of nitration of CCL2 *versus* CXCL12.

CCL2 is a chemoattractant factor produced by multiple cell types, including tumors. Activated cluster of differentiation (CD)4^+^, CD8^+^, natural killer, and immunosuppressive myeloid cells respond to CCL2 gradients and localize to sites of insult or injury. Immune cells use cytokine and chemokine gradients to track toward inflammation, infection, or tumor sites where they execute their functions to maintain homeostasis. Nitration on tyrosine 13 decreases CCL2 affinity for its receptor, CCR2 (163), but has little effect on binding to glycosaminoglycans (161). T-cells, which express fewer CCR2 receptors compared to immunosuppressive myeloid cells, become unresponsive to high levels of nitrated CCL2, while myeloid cells continue to respond, although at higher CCL2 concentrations. Thus, nitration of CCL2 leads to selective enrichment of immunosuppressive myeloid cells at inflammatory sites in tumors, and relative reduction of T-cells, thus providing a tumor-permissive microenvironment.

Similarly, the effect of CCL2 in recruiting inflammatory immune cells to a murine artificially introduced synovium-like air pouch was demonstrated. In the murine air pouch model of inflammation, air is introduced subcutaneously in the back of mice over several days to disrupt the interface between skin and connective tissue, generating a fluid filled pouch of inflammatory exudate (164). Unmodified CCL2 injected into mice resulted in the recruitment of leukocytes into the air pouches, while injection of nitrated CCL2 into the air pouches did not recruit inflammatory leukocytes and systemically administered nitrated CCL2 blocked recruitment of proinflammatory leukocytes to CCL2-treated air pouches (163). This leads to selective enrichment of myeloid cells at inflammatory sites, in tumors, and the tumor microenvironment, and a comparative decrease in CD8^+^ T-cells (161). Nitration directly decreases the ability of CCL2 to recruit cytotoxic CD8^+^ T-cells to tumor sites, while immunosuppressive cells such as MDSC with higher CCR2 expression may still respond to the modified chemoattractant. The discrepancy between these studies is yet to be resolved and will provide important understanding in the role of CCL2 nitration in leukocyte trafficking and cancer growth.

T-cell recruitment to the tumor microenvironment and inflammatory sites is hampered by nitration, while effects on myeloid cells, including immunosuppressive MDSCs, is either reduced or minimally affected. MDSCs can produce peroxynitrite extracellularly resulting in the modification of target cell proteins and has been reviewed previously (165, 166). In addition to inhibiting T-cell recruitment through chemokine nitration, MDSCs can also inhibit T-cell function through the nitration of the T-cell receptor antigen binding domains (167). This destroys antigen recognition and renders the modified T-cells unresponsive to antigen binding and activation (168). Blocking CXCR4, the receptor for CXCL12, with plerixafor (AMD-3100) increased the effectiveness of neogenesis blood vascular disrupting agent combretastatin A4 nanodrug (169) in a mouse model of breast cancer, presumably by preventing immunosuppressive macrophages from being recruited to tumors. These data suggest that selective nitration of CXCL12 is a mechanism by which chemotaxis is regulated under pathological conditions and could be important for immune surveillance in oncology.

## Nitration of PECAM-1 (platelet endothelial cell adhesion molecule) and chemotherapy resistance

PECAM-1 is a cellular adhesion molecule that is expressed on the surface of platelets, endothelial cells, and leukocytes, as well as on the surfaces of solid tumor and lymphoid cancer cells. The protein, which has an established role in angiogenesis, leukocyte migration thrombosis, and plaque formation, is regulated by phosphorylation on its cytoplasmic domain on two immunoreceptor tyrosine-based inhibition motifs in its cytoplasmic domain. Phosphorylation of these sites serve as SHP2/SHIP-2 docking sites and promotion of signal transduction. Nitration of the intracellular domain of PECAM-1 inhibits the binding of protein tyrosine kinases (SHP2) and phosphatases (SHIP-2) that regulate cell growth (104). Disrupting PECAM-1 signaling may thus alter the tumor microenvironment to promote tumor proliferation and support late-stage metastasis (170). PECAM-1 has also been implicated in resistance to Etoposide, a widely used chemotherapeutic that induces double-strand breaks in DNA to cause cells to stall their cell cycle at the entry to mitosis and subsequently undergo apoptosis. Overexpression of PECAM-1 in cells that normally lack the protein reduces sensitivity to etoposide-induced apoptosis. In addition, shRNA knockdown of PECAM-1 in leukemia cells causes increased sensitivity to the therapeutic (152). More work is needed to understand the role and therapeutic targeting potential of tumor-expressed PECAM-1.

## ER stress/damage-associated molecular patter expression

HSPs, PDIs, and high-mobility group proteins have roles in protein folding quality control *via* the unfolded protein response. Nitration of HSP90 on tyrosine 33 (Y33) and tyrosine 56 (Y56) results in PC12 neuronal cell death (171). Site-directed mutagenesis of Y33 and Y56 on HSP90 to phenylalanine protects PC12 cells from peroxynitrite-induced death (171). Mechanistically, nitration of Y33 decreases mitochondrial activity, whereas nitration at Y56 induces cell death in a P2X7R-PTEN dependent manner (84, 172). Increased levels of nitrated Y56 on HSP90 have been shown in both mouse models of amyotrophic lateral sclerosis and spinal cord trauma as well in motor neurons from amyotrophic lateral sclerosis patients, suggesting that therapies targeting nitrated HSP90 merit investigation. In contrast, in merlin-deficient schwannoma cancer cells, increased nitrated HSP90 is believed to contribute to the Warburg effect by decreasing mitochondrial oxidative phosphorylation and increasing dependance on glycolysis and glutaminolysis (173). Treatment of these cells with peroxynitrite scavengers significantly decreases survival, although the role of nitrated HSP90 in this phenotype was not well established. Cellular context may play a dramatic role on the functional consequences of pathologic HSP90 nitration, as illustrated in these two models.

PDIA3 (ERp57) is normally found in the ER where it has an essential role in protein folding through formation of disulfide bonds. It functions in the unfolded protein response by rearranging misfolded proteins to alleviate ER stress and promote cell survival (174). Nitration on tyrosine residues at positions 67 and 100 (50) has been reported and nitrated PDIA3 is associated with mitochondrial disease (175). Membrane-associated PDIA3, which is overexpressed in most cancer types, is prognostic in various cancers and is especially expressed in malignant cells and monocytes/macrophages. It is significantly correlated with immune-activated hallmarks, cancer immune cell infiltrations, and immunoregulators, and could significantly predict anti-programmed death-ligand 1 therapy response (176). Knockdown of PDIA3 significantly weakens the proliferative and invasive ability of glioma cells and PDIA3 deletion suppresses mitochondrial activity in human cerebral microvascular cells through increased phosphorylation of STAT3 (177). It may also have a role in immunogenic cell death through its association with calreticulin on the cell surface as an early marker of apoptosis (178). Further experiments are needed to determine the effect of nitration on PDIA3 in cancer.

## Nitration of HDAC2 and regulation of gene expression

Nitrated proteins with roles in regulating gene expression have also been identified. Histone deacetylase 2 (HDAC2), a known anticancer therapeutic target, has been shown to be inhibited by nitration. Specific nitration of HDAC2 on Y253 results in ubiquitinylation and subsequent proteasomal degradation, resulting in lower levels of the protein (179). Inhibition of HDAC function allows genes repressed by histone deacetylation to be expressed thus supporting anticancer activity. Similarly, SIRT2 and SIRT6 are inactivated by nitration (Y86 and Y257, respectively) which leads to apoptosis and inflammation. The splicing factor SF3A2 regulates gene expression through the proper processing of RNA transcripts. It has also been shown to be associated with the mitotic spindle and required for proper chromosome segregation (180). Deletion or antibody blockade of SF3A2 leads to mitotic arrest and eventual triggering of apoptosis. The role of SF3A2 nitration has not been documented and a description of the effect on this critically functioning mitotic regulator will be important to understand.

## Summary and outlook

In this review, we provide a new resource, an updated and curated database of human tyrosine nitrated proteins, and a summary of insights into the roles of nitration in oncology. As with tyrosine phosphorylation, certain caveats with the interpretation of the data exist, specifically those related to MS-based identifications. For example, an estimated 26,000 tyrosine phosphorylation sites identified in PhosphoSitePlus may be false-positive identifications (181) because of inaccurate localization of tyrosine phosphorylated sites on peptides by MS-platforms (182). Similar concerns, which have been discussed previously, exist for tyrosine nitration (56, 59). Another caveat is that our meta-analysis includes putative sites of nitration identified by exposing proteins, cells, or tissue to *in vitro* nitration. Like tyrosine phosphorylation a dynamic process controlled by the actions of kinases and phosphatases, similar process may govern the detection of tyrosine nitrated proteins. As such the detected nitrated proteins may reflect longer lived and more stable sites of modification. Despite all these caveats, phosphorylation and nitration of tyrosine residues may represent overlapping or competing functional regulatory PTMs in the proteome.

Interrogation of this database has unveiled several forward thinking possibilities that could fuel future research.

1. Tyrosine nitration may play a functional role in protein transcription, translation, and ER processing.
2. Nitration of molecular chaperones and chaperonins that facilitate proper mitochondrial import of proteins and assembly of ETC protein complexes may regulate mitochondrial bioenergetics and function.
3. Despite several experimental evidence indicating that tyrosine nitration is a dynamic process, enzyme(s) that specifically remove the nitro group from tyrosine nitrated proteins have not been conclusively identified.
4. As with other tyrosine PTMs, which are catalyzed by specific enzymes, nitration is substoichiometric and specific for certain tyrosine residues within proteins. Thus, nitration of protein tyrosine residues may also by catalyzed by specific enzymes. Several reports demonstrate that enzymatic catalysis of protein nitration is possible. Proteins with redox active metal centers can catalyze their own site-specific nitration (1, 10, 11, 12, 13, 14, 15) and possibly that of other proteins as suggested for SOD 1 (1, 4). Peroxidases such as MPO and EPO catalyze nitration of proteins in inflammatory conditions (6, 7, 8, 9). However, the level of the overlap between tyrosine nitration and phosphorylation indicates that specificity could be driven by additional enzymatic sources. We envision a class of enzymes termed nitrases, similar to tyrosine kinases, each of which will target specific proteins and execute site-specific nitration. Structural analysis of the tyrosine residues that can be both phosphorylated and nitrated could reveal new insights for the biochemical and biophysical properties that govern site specific nitration of tyrosine residues in proteins and facilitate the discovery of nitrases.
5. Functional regulation and aberrant signaling through tyrosine nitration and phosphorylation may contribute to oncogenesis, tumor biology, and to the immune responses to cancer suggesting that identification of specific nitrases enzymes could provide prominent new therapeutic targets.

In conclusion, we suggest that future study of protein nitration and its enzymatic regulation has the potential to open new areas of enzymology, biology, and drug discovery, with important implications for disease areas that include oncology, neurodegenerative disease, and autoimmunity.

## Supporting information

This article contains supporting information.

## Conflict of interest

I. G.-P., A. K. K., S. M., Z. W. H., and H. I. are option holders in Nitrase Therapeutics. The author, H. F. declares no conflicts of interest with the contents of this article.
